# Complementary Analysis for Undetectable Microplastics from Contact Lenses to Aquatic Environments via Fourier Transform Infrared Spectroscopy

**DOI:** 10.3390/molecules28093713

**Published:** 2023-04-25

**Authors:** Jieun Lee, Yejin Lee, Jeonghyeon Lee, Minseong Kang, Sanghyun Jeong

**Affiliations:** 1 Institute for Environmental and Energy, Pusan National University, Busan 46241, Republic of Korea; 99atkins07@pusan.ac.kr; 2 Department of Environmental Engineering, Pusan National University, Busan 46241, Republic of Korea

**Keywords:** microplastics, Fourier transform infrared spectroscopy, plastic waste, contact lens, nanoplastics

## Abstract

Although microplastics (MPs) are intrinsically toxic and function as vectors for organic micropollutants, their discharge from wastewater treatment plant effluents and human activity remains unknown owing to the limitations of detection and treatment technologies. It is imperative to quantify MPs from human activities involving the consumption of various plastic products. This study warns that contact lenses can generate MPs and nanoplastics (NPs) after being discharged into aquatic environments. Identification via micro-Fourier transform infrared spectroscopy revealed that the fragmented particles (from a few tens to a few hundred micrometres) could not be detected as poly(2-hydroxyl methacrylate), the component of contact lenses, owing to changes in its chemical properties. After the degradation process, the median size of the contact lens particles decreased from 313 to 85 µm. Approximately 300,600 g of contact lens waste is discharged into sewage systems daily in the United States of America (USA), where 45 million people wear contact lenses and throw away one-fifth of them every day. Contact lens waste (1 g) has the potential to release 5653.3–17,773.3 particles of MPs. This implies that the currently reported MP amounts in the environmental matrix exclude significant amounts of MPs and NPs from discharged contact lenses. The identification method should be examined, and a registration of the disposal process should be established.

## 1. Introduction

With the increasing use of plastic products by human activity, natural environments are becoming increasingly exposed to discharged microplastics (MPs). For instance, MPs have been detected in ecosystems [1,2,3] as well as in food items such as fish [3,4], salt [5,6], seaweed [7], and seafood [8]. These MPs may have originated from wastewater treatment process (WWTP) effluents and the indiscreet disposal of plastic waste into the ocean and soil environments [9,10,11]. Although more than 90% of MPs are removed by conventional treatment processes, 10% of unretained MPs and undetectable or untreatable NPs are released from WWTP into aquatic environments.

In addition to WWTPs as MP/NP sources, recent studies have revealed that plastic products used in human activities release considerable amounts of MPs into the environment: 140 ± 19–6292 ± 10,521 particles/L from one water bottle [12,13,14], 11.6 billion particles/L from one teabag [15], and 693–1066 particles/L from one wet wipe sheet [16]. Moreover, silicone rubber and commercial facial scrubs can generate MPs [17,18].

The discharge of MPs from WWTPs and human activities has a secondary issue: NP generation after exposure to environments under weathering conditions (erosion, ultraviolet degradation, etc.) [10,19,20]. In particular, the negative impacts of NP health risks, including potential toxicity to microorganisms and the human body [21,22], are emerging concerns. MPs and NPs can enter the human body through the intake of water, beverages, and other food sources [23,24] and may accumulate in the body [25]. A recent study reported that polystyrene NPs can induce structural deformation in human serum albumin [26]. In addition to their intrinsic toxicity, NPs are vectors for transporting trace amounts of organic contaminants via hydrophobic interactions with large adsorption areas [27,28].

The quantification/qualification of MPs in an environmental matrix have been conducted using spectroscopy, which can identify hydrocarbons based on their unique fingerprints from the absorption of incident infrared (IR) light. Fourier transform infrared (FTIR) is the most widely used analytical method for identifying MPs above 30 µm [29,30,31]. Owing to its non-destructive and accurate detection of trace amounts of MPs, FTIR with chemical imaging analysis is mainly applied for MP detection in surface water and treated water during the water treatment process [32,33]. Recently, an application of near-infrared (NIR) hyperspectral imaging and Raman spectroscopy to MP identification increased the detectable particle size range between 1 and 50 µm [34]. To establish the standardisation of MP identification, the detectable particle size range and improvements in the accuracy of spectroscopic methods should be systematically examined in various types of environmental matrices that can underestimate the actual MP amounts by interfering with their IR spectra.

The daily use of contact lenses has continued to increase owing to their convenience. Despite this increase, their indiscriminate disposal into aquatic environments through toilets and sinks has not been considered of great importance as an MP source in the environment. Their inappropriate disposal in aquatic environments can be a significant source of MPs/NPs transferred to the environment and, subsequently, to the human body. Plastics are non-degradable and have remained in the environment for at least 100 years. Therefore, they have strong and long-lasting effects on the environment after disposal. However, to date, no studies have performed qualitative and quantitative analyses of the MPs released from contact lenses into aquatic environments after discharge.

This study examined the generation of MPs from contact lenses discharged and released into aquatic systems. The experiments were designed based on the pathway of exposure to the environment. The MPs generated according to the exposure scenario were analysed spectroscopically using micro-FTIR. The identification of MPs using micro-FTIR was investigated to determine whether they can be detected and quantified; this method can be applied for MP monitoring in drinking water treatment plants (DWTPs), WWTPs, and in laboratories for MP detection in daily products.

## 2. Results

### 2.1. Method Validation of the Released MPs from Contact Lenses

**Figure 1:**
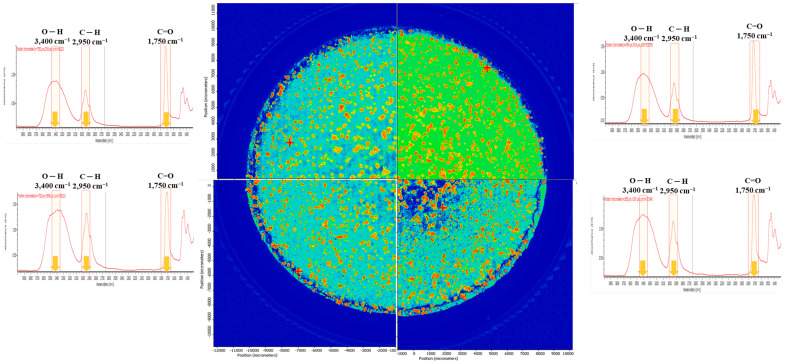
Identification of PHMA reference using chemical image mapping.

For the method validation of MP identification in the MPs release test, the poly(2-hydroxyethyl methacrylate) (PHMA) reference material was dispersed in deionised (DI) water, and the identification with automatic image analysis was assessed based on the previous study [35]. Figure 1 shows the chemical image mapping of the PHMA reference on the Anodisc filter. Each particle of the PHMA was clearly distinguished. A broad C–H peak at 2950 cm^−1^, a broad hydroxyl (-OH) peak at 3400 cm^−1^, and a sharp carbonyl (C=O) peak at 1750 cm^−1^ were observed in the IR spectrum of PHMA in DI water. A recovery rate above 70% was assumed to be reliable and can be applied for MPs identification. The recovery of the PHMA fragments that was detected using current analytical methods, the automatic image analysis of micro-FTIR, was above 91.12 ± 0.27%.

### 2.2. Identification of Contact Lenses: Chemical Properties

#### Qualitative Analysis of Unused Contact Lenses

**Figure 2:**
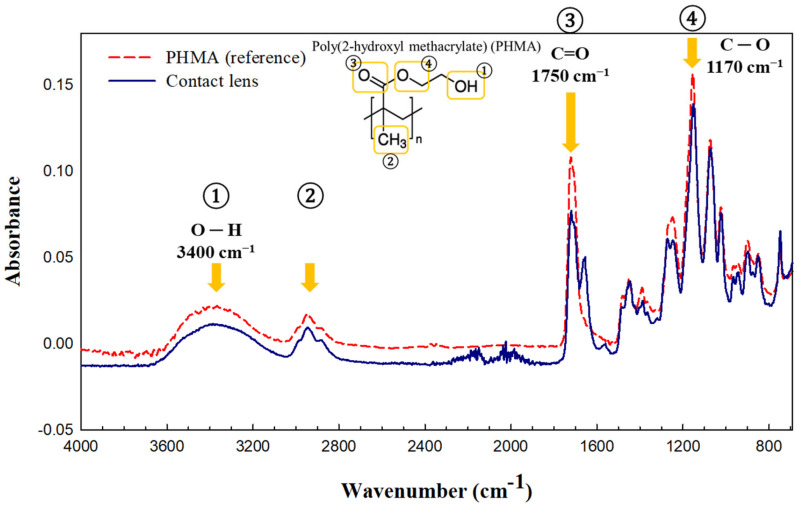
Fourier transform infrared spectroscopy (FTIR) spectrum of the contact lens acquired in the attenuated total reflection (ATR) mode, compared with the reference of chemical structure.

The chemical compositions of the contact lenses were analysed in both attenuated total reflection (ATR) and transmission modes of the micro-FTIR. The IR spectrum of the dried contact lens particles was compared with that of the poly(2-hydroxyethyl methacrylate) (PHMA) referenced in Figure 2. The PHMA reference has a broad C–H peak (②) at 2950 cm^−1^, a broad hydroxyl peak (-OH) (①) at 3400 cm^−1^, a sharp carbonyl (C=O) peak (③) at 1750 cm^−1^, and another sharp peak for (C–O) at 1170 cm^−1^, which represents the molecular functional group in the chemical structure of PHMA (inset in Figure 1). The IR spectrum of the contact lens (dark blue line) matches that of the PHMA reference (red dashed line), as shown in Figure 1. It was also identified as PHMA with 87.71 scores out of 100 based on matching with the reference library [Sprouse polymers by ATR, OMNIC software (Thermo Scientific™ OMNIC™ Picta™), Thermo Fisher Scientific]. PHMA as the host material and poly(methyl methacrylate) (PMMA) for increasing hardness are common materials used for manufacturing soft contact lenses [36,37]. N-vinyl pyrrolidone (NVP) in poly(NVP-co-HMA) hydrogels is used as a soft contact lens material. PHMA is an inert, water-stable, and non-degradable hydrogel with high transparency [38]. Their physical properties can be easily modified by changing the crosslinking density and copolymerisation [39]. As shown in Figure 1, PHMA contains hydrophilic functional groups (carboxyl C=O and -OH groups). It is used in contact lenses because of its physicochemical characteristics.

### 2.3. Release of MPs from Contact Lenses to Water

#### 2.3.1. Qualitative Analysis of the Fragmented Contact Lens after Usage

Usually, contact lens users take out their lenses, rub them off with their fingers, and finally throw them into the toilet, basin, or trash. During disposal after use, the contact lenses are folded or stuck together, resulting in multiple layers of folded contact lens fragments. This means that a single layer of contact lens fragments generated after disposal is hardly present in real-life situations. We identified several contact lens particles prepared using the same disposal procedure. The qualitative/quantitative analysis of the MPs released from the contact lens fragments followed the same analytical procedure used in the DWTPs and the laboratory for monitoring MPs released from daily products.

**Figure 3:**
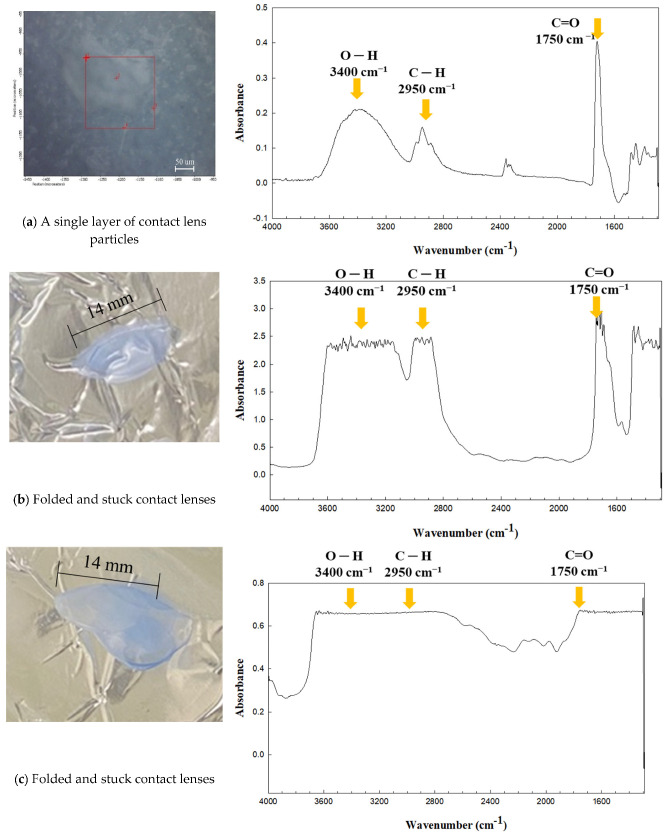
Infrared (IR) spectra of the contact lens particles that were dried under a desiccator: (**a**) one layer (red square: aperture), (**b**) two layers, and (**c**) multiple layers of folded contact lenses. IR spectra were acquired in the transmission mode of the micro-FTIR.

However, the contact lens fragments in the MP release test did not show an appropriate IR band corresponding to the functional groups of the molecular structure of the dried contact lens (Figure 2). Figure 3 shows the IR spectra of the three contact lenses with different layers acquired in transmission mode. As shown in Figure 3b, folded lenses with two layers showed no distinctive IR peaks in the contact lenses, in which IR bands were observed at the representative wavenumbers shown in the reference PHMA (Figure 2). As the thickness of the attached contact lenses increased (Figure 3b,c), the representative peaks became indistinguishable and disappeared (Figure 3c). The line-shaped distortion in the IR spectrum indicated that spectra (b) and (c) were saturated. This is presumably due to the changes in the thickness of the contact lenses, and the transmittance and absorption are affected by the material [40].

#### 2.3.2. Identification of the MPs Released from Fragmented Contact Lenses after Usage

In a contact lens release test set up as described in Section 3.2, MPs were released from the contact lens fragments into the liquid. The MPs in the liquid were collected on an Anodisc filter and identified using micro-FTIR for automatic analysis. The MP release test mimicked MP generation during the contact lens disposal procedure, and MP identification followed the same procedure as that in the DWTPs and laboratories for MPs released from daily products.

**Figure 4:**
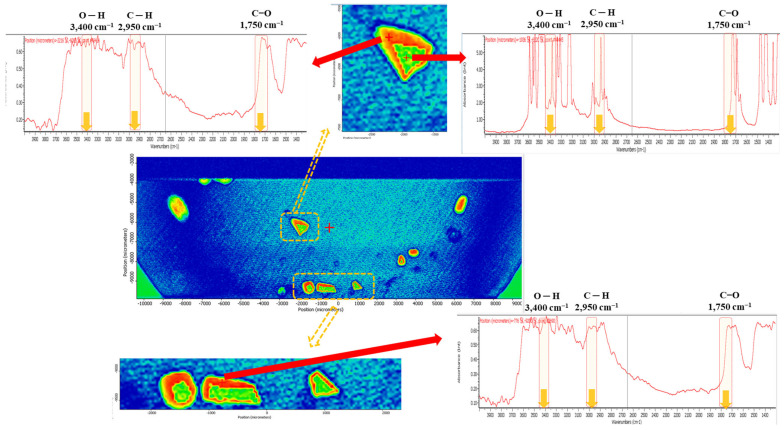
Chemical image mapping of the contact lens fragments in DI water, corresponding to the condition when contact lens fragments are discharged into a toilet/basin (aquatic environments). IR spectra were acquired in the transmission mode of the micro-FTIR.

The MP fragments in the released simulants exhibited no characteristic absorption bands corresponding to the functional groups of the PHMA reference (Figure 4). As shown in the chemical image (Figure 4), the IR bands of each MP fragment were saturated owing to the thickness of the fragments, which were folded into several layers. The identified fragments are presented with different false colours that represent the hierarchy of correlation with the PHMA reference. The presence of several folded layers is inevitable when lenses are used and disposed of in toilets or basins. This led to the failure of particle identification as a PHMA MP because of the saturated IR band. This indicates that during the quantitative/qualitative analysis of MPs in water using micro-FTIR, MPs released from the discharged contact lenses are undetected and not counted in the total amount of MPs when applying the MP analytical procedure in the WTP and laboratory for monitoring MPs released from daily products. ATR-FTIR analysis is recommended for the identification of contact lens fragments generated during the disposal process.

#### 2.3.3. MP/NP Release by Degradation

**Figure 5:**
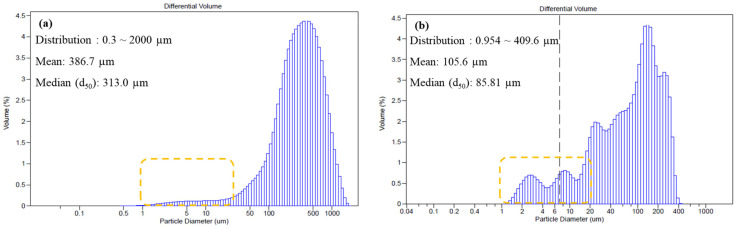
Size distribution of contact lens particles (**a**) that emerged in deionised water and (**b**) after chemical degradation via HCl treatment.

Contact lens waste fragments can be transported to aquatic environments and generate NPs for long periods of time under degradation conditions. For harsh conditions of decomposition, the as-prepared contact lens fragments were exposed to a 3% hydrochloric (HCl) solution (in DI water). HCl treatment can change the chemical properties of PHMA, resulting in the misidentification of MPs using micro-FTIR. Thus, the presence of NPs was examined by measuring the particle size distribution of the MP fragments subjected to degradation conditions. The initial particle size was controlled by hand grinding and varied between 2000 and 0.3 µm (Figure 5a). As a control, the ground contact lens particles were immersed in DI water, and the particle size distribution was measured using PSA via the laser diffraction method. No change was observed in the particle size distribution, d_50_, which represents the median value, and it did not change during the experimental period (seven days) (Figure 5a). In the degradation experiment, MP generation from contact lens particles in aquatic environments was simulated by immersing contact lens particles in 3% HCl solution for one day. In contrast to the control, after degradation with HCl, the particles fragmented into smaller particles. The mean value decreased from 386.7 µm to 105.6 µm, classified as MPs. The median (d_50_) decreased considerably from 313.0 µm to 85.81 µm. The large particle size distribution decreased after degradation (Figure 5a,b). While the initial volume of the large particle size distribution between 400 and 2000 µm disappeared after chemical degradation, that of the small particle size distribution of <20 µm increased (highlighted by the yellow dashed line). This indicates that the larger particles fragmented into smaller particles.

Considering the definition of MPs (plastic particles from 1 mm up to 1 µm), the contact lens fragments before/after degradation are classified as MPs. The degradation process accelerated MP generation via fragmentation into a smaller size (<20 µm, highlighted to yellow frame in Figure), which is also not detectable by commonly applied MP analysis—vibrational spectroscopy (m-FTIR and m-Raman). This implies that NP generation can be accelerated by inducing MP fragmentation under degradation conditions.

**Figure 6:**
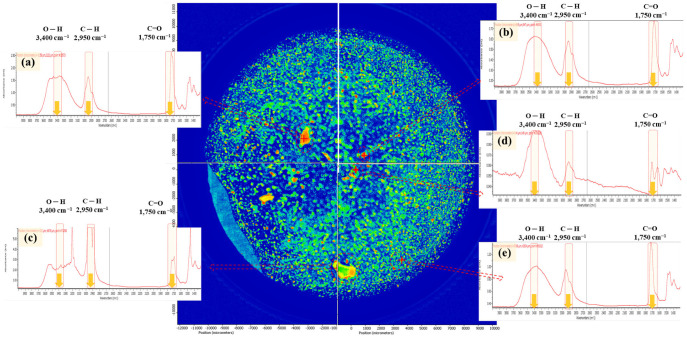
Chemical image mapping of MPs released after long-term exposure: (**a**,**b**,**e**) IR spectra of PHMA MPs; (**c**) IR spectrum of multi-layered contact lens fragments; and (**d**) IR spectrum of contact lens fragments with a low matching score (0.3–0.6 out of 1).

To quantify the MPs generated after long-term exposure to aquatic environments, an MP release test was conducted for 1 year. The amount of MPs released into the DI water from the contact lens fragments is shown in Table 1. Seven to ten pairs of contact lens fragments released 1696–5332 MPs, the size of which was in the 20–300 µm range. MPs below 100 µm accounted for more than 70% of the total MPs, while 50% of fragments were more than 386.7 µm in size in the initial state. These results indicate that fragments of contact lens waste can generate MPs below 100 µm under long-term exposure to aquatic environments. Meanwhile, 0.6–2.3% of contact lens fragments were undetectable. In the IR spectrum of the fragments, saturated peaks from the thickness (Figure 6c) and the disappearance of the IR bands [corresponding to a low matching score (0.3–0.6 out of 1)], probably due to chemical degradation (Figure 6d), were observed (Figure 6). These undetectable MP fragments freely pass through MP monitoring in WWTPs and DWTPs.

### 2.4. Environmental Implications

#### 2.4.1. Generation of MPs

During the disposal process of contact lenses into wastewater systems, fragmentation of contact lenses and their discharge into the sewage system were observed, generating small plastic particles in the range of 400 mm to sub-micrometres, the majority of which are classified as MPs. This indicates that the fragmentation of contact lenses during disposal can release MPs into aquatic environments.

#### 2.4.2. NP Generation: Are They Undetectable?

Particle sizes down to 0.9 µm indicate NP generation after being submerged in water and degraded by UV and/or acidic conditions, and fragmentation of contact lenses may generate NPs [41,42]. To date, NP identification using current analytical technologies (FTIR microscopy and micro-Raman) is limited; thus, their rejection efficiency cannot be assessed in effluents from WWTPs or DWTPs [43]. This means that considerable but unknown amounts of NPs are freely discharged into aquatic environments and are further transferred to soil and microorganisms. NP generation is accelerated after exposure to weathering conditions [44,45].

#### 2.4.3. Hazardous Effect: Transfer of Pollutants to the Human Body, Intrinsic Toxicity

Studies have reported considerable protein build-up on contact lens surfaces [46]. This indicates that the fragmented contact lens could act as a vector for organic contaminants and microorganisms. The exposure potential can be accelerated even when the size of the particles decreases owing to weathering processes (erosion, UV degradation, and biological/chemical decomposition) after long-term exposure to aquatic environments. MPs are composed of hydrocarbons and thus intrinsically have a high affinity for organic contaminants via hydrophobic interactions. The fragmentation of NPs can induce an affinity for organic contaminants owing to their increased effective surface area [47,48]. Thus, their ability to transfer to microorganisms and further to the human body via the food web is accelerated when their size decreases to the NP range (down to 1 µm) [24]. However, PHMA contains a 2-hydroxyl functional group on one molecule, indicating that its surface is hydrophilic. It also induces interactions with proteins owing to its ionic affinity [49,50].

In addition to their role as vectors for pollutants, MPs contain intrinsically toxic substances (plasticisers, unreacted monomer residuals, etc.) that are added during synthesis in the manufacturing step [51]. For example, the release of phthalate esters causes adverse effects in the mouse gut [52]. These chemicals have been reported to have harmful effects on microorganisms and potentially on the human body [53].

#### 2.4.4. Daily Discharged Amounts to the Environment but Undetected by the Currently Used Method (micro-FTIR)

Even though significant amounts of contact lenses are discharged into aquatic environments and generate MPs, further generating NPs after long-term exposure to degradation, these MPs/NPs are undetected by the current identification method. MPs collected on the filter after sample pre-treatment were scanned by micro-FTIR in transmission mode, and the particles identified as MPs after correlation with the reference are presented as a chemical image map [29,30,54]. In mapping, where a large spatial area is automatically scanned, the transmission mode is applied rather than the reflection and ATR modes. However, because of the absence of characteristic absorption bands of the functional groups of PHMA after degradation and saturated peaks due to the thickness of the contact lens fragments, MPs released from contact lens waste were undetected using micro-FTIR in transmission mode.

We estimated the amount of contact lens waste generated by users daily in the USA. In the USA, 45 million people wear contact lenses, and one-fifth of contact lenses are flushed into toilets and/or thrown into basins after daily usage. This corresponds to approximately 10 metric tonnes of MPs released into wastewater treatment plants and aquatic environments [55]. The detailed procedure for estimating the amount of contact lens waste is presented in Table 2. Based on the measured weight of an individual contact lens (0.0167 ± 0.0004 g), 300,600 g of contact lenses were estimated to be disposed of in the sewage system. After the MP release test, 7–10 pairs of contact lenses generated 1696–6.176 particles of MPs. This corresponds to 655.89–2246.06 p/g of contact lens waste every day.

Recent studies have reported that MP amounts from WWTP effluents, natural surface water, and oceans range from a few tens to a few thousand particles per litre [32], excluding the unknown number of MPs released from 300,600 g of contact lens wastewater per day.

However, owing to the limitations of MP detection in waste contact lens particles, MPs released from contact lenses pass through regular MP monitoring in WWTPs and DWTPs. Furthermore, the NPs fragmented from the contact lens particles were not retained in the current treatment process because their size (1 µm) was smaller than the porosity of the sand filtration system (approximately 20 µm). Consequently, unknown amounts of undetectable MPs released from contact lens waste are discharged into aquatic environments. A detailed procedure for identifying contact lens particles should be formulated. Furthermore, a definition, a proper disposal process, and public education on MPs released from contact lenses should be established.

## 3. Materials and Methods

### 3.1. Materials

Two commercially available (disposable) contact lenses produced by two companies (A and B) were selected for this experiment. Before the test, the contact lenses were dried in a desiccator at 25 °C to remove any water covering them. To simulate the conditions of discharge into the environment, the as-dried contact lenses were fragmented into small sizes by grinding them with a mortar and pestle.

### 3.2. Experimental Design for MP Generation from Contact Lens Discharge

**Figure 7:**
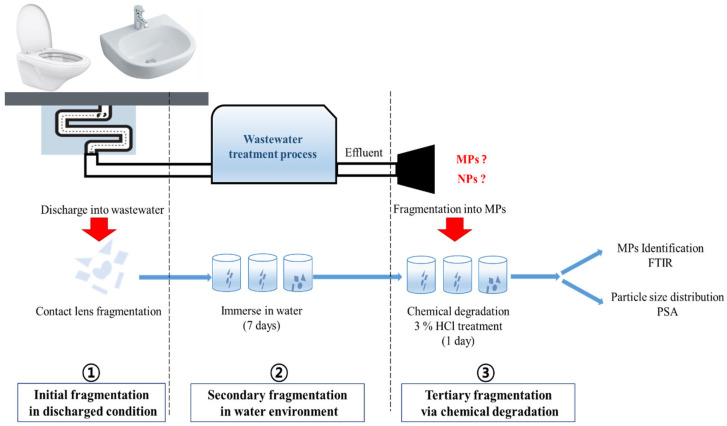
Schematic of microplastic (MP) and nanoplastic (NP) release tests based on the disposal process of contact lenses (①→②→③).

MPs can be released from contact lenses in three fragmentation steps when contact lenses are used and discharged. ① Used contact lenses are generally rubbed off by fingers and thrown into toilets or sinks, from where water is transported to the WWTP. The contact lenses were fragmented into small pieces. ② During their transportation and primary treatment in the WWTP, contact lenses can be easily fragmented into smaller particles. We simulated this scenario to prepare the samples and perform an MP release test (Figure 7). To prepare the contact lens samples without liquid for preservation, new contact lenses were removed from the cases and dried in a vacuum desiccator until they exhibited a constant weight. To mimic the rubbing process of the used contact lenses (from ①) that fragmented into small pieces, the as-dried contact lenses were ground using a mortar and pestle. For process ②, the fragments were immersed in water for seven days to simulate the retention time of the discharged wastewater passing through the sewage system and the WWTP. ③ Then, to accelerate the fragmentation into MPs (chemical degradation), the sample was immersed additionally for one day in a 3% hydrochloric acid (HCl) solution with deionised (DI) water. After the release test, the homogenised liquid with contact lens particles was aliquoted for the qualification and quantification of MPs released from the contact lenses via micro-FTIR analysis.

The sample preparation procedure mimicked the actual conditions of contact lens disposal after usage. The initial fragmentation under the discharge condition (①, Figure 7) is shown in Table 3.

### 3.3. MP Identification via FTIR

The composition of the contact lens was determined using FTIR spectroscopy (Nicolet iN10, Thermo Fisher Scientific, USA) in attenuated total reflection (ATR) mode. For sample preparation in the ATR mode, the dried contact lenses were fragmented into small pieces, and heterogeneously sized contact lens particles were placed on a slide in contact with the ATR tip. The infrared (IR) spectra were obtained for wavenumbers ranging from 500 to 4000 cm^−1^ with 16 scans and a resolution of 4 cm^−1^.

For the analysis of MPs released from the contact lens particles, the as-taken contact lenses were treated with 3% hydrogen peroxide (H_2_O_2_) to remove any additives in the preserving liquid that could block the original IR signal of the contact lens. The treated MPs in the liquid were collected and filtered through an Anodisc filter (Al_2_O_3_, diameter: 25 mm, pore size: 0.2 µm, Cytiva Whatman, Marlborough, MA, USA) using vacuum filtration.

Particles suspected on the Anodisc filter were identified using micro-FTIR in transmission mode (Nicolet iN10 and iN10MX, Thermo Fisher Scientific, Waltham, MA, USA). This methodology is based on a combination of chemical imaging and point analysis using a mercury cadmium telluride (MCT) detector.

For chemical image mapping, an array detector that can analyse a 25 mm filter area by scanning an array of 16 sectors simultaneously collects the spectral information of heterogeneous particles. The spectra of the particles on the Anodisc filter were acquired for wavenumbers ranging from 1300 to 4000 cm^−1^ with a 3 s collection time (16 scans), 60 µm × 60 µm aperture size, and 8 cm^−1^ resolution. The wavenumber below 1300 cm^−1^ was subtracted because aluminium oxide (Al_2_O_3_), a component of the Anodisc filter, is transparent to IR radiation above 1300 cm^−1^ [56]. After chemical image mapping, the datasets were correlated with the IR spectrum of the contact lens composition. One quarter of the filter area was individually analysed, and the data from each quarter were summed.

For single-point analysis using an MCT detector, the spectrum of a single particle in the range of 1300–4000 cm^−1^ was acquired with a collection time of 1 s (eight scans), a 50 × 50 aperture size, and 8 cm^−1^ resolution. MPs identification was conducted by visually counting the proposed particles that correlated to more than 60% of the contact lens after image analysis with micro-FTIR.

### 3.4. Particle Size Distribution

The particle size distribution profiles of the fragmented contact lenses were examined using a particle size analyser (PSA, LS 13 320, Brea, CA, USA) based on the laser diffraction method. Eighteen dried contact lenses were physically fragmented and immersed in water under the same release conditions, as shown in Figure 5. After immersion of the contact lens fragments in DI water for five days and chemical degradation for one day to accelerate MP fragmentation, 5 mL of liquid containing the fragments of contact lenses was aliquoted, and injection of 5 mL of the sample into the sample port of the PSA was performed until it met the detection limit.

## 4. Conclusions

Generally, in transmission mode, the qualification and quantification of MPs in water are conducted using micro-FTIR because it enables the automatic scanning of all suspected particles collected from the bulk matrix in the most efficient measurement times. The MP amounts in the environmental matrix were first identified by correlation with reference MPs, and the as-identified particles were counted as MPs. However, in this study, it was difficult to detect MPs released from contact lenses discharged into the water and soil environments, and these MPs were undetectable by the current analysis. Fragments of contact lens waste were difficult to identify using micro-FTIR because its irregularly formed layer (single and multi-layer) was inevitably formed during the disposal process by users.

Considerable amounts of MPs/NPs have been omitted from the currently reported MP amounts in aquatic environments. Considering the daily consumption of contact lenses (300,600 g) in the USA, considerable amounts of MPs are generated and discharged into the environment. However, these concentrations are unknown because MPs are undetectable using current analytical methods. Further studies should focus on accurate analysis and the establishment of a disposal treatment.

## Figures and Tables

**Table 1:** Quantification of MPs released from contact lens fragments after a release test for 1 year using chemical image mapping of micro-FTIR analysis. (a) Contact lens A; and (b) Contact lens B.

(a) Contact Lens A	Detected MPs		Undetected MPs	
	Number of MPs	Proportion (%)	Number of MPs	Proportion (%)
**Total**	1696	97.70	40	2.30
**>100 µm**	484	27.88	32	1.84
**50–100 µm**	792	45.62	8	0.46
**20–50 µm**	420	24.19	0	0.00
**(b) Contact Lens B**	**Detected MPs**		**Undetected MPs**	
	**Number of MPs**	**Proportion (%)**	**Number of MPs**	**Proportion (%)**
Total	5332	99.26	32	0.60
>100 µm	2628	48.99	32	0.60
50–100 µm	1792	33.41	0	0.00
20–50 µm	912	17.00	0	0.00

**Table 2:** Estimation of the amount of contact lens waste in the USA and MP release.

		Contact Lens Waste (g/Day)	Number of MPs Released from Contact Lens Wastes (Particle/g of Contact Lens·Day)
Estimation	Weight of individual dried contact lens (g)	(0.0167 ± 0.0004) g	
	People wearing contact lenses	45 million people	
	Number of contact lenses discharged into toilet/basins	One fifth	
	Formula	(45 million × 1/5) × (0.0167 × 2) g	
Estimated results		300,600 g/day	5653.3–17,773.3 p/g·day

**Table 3:** The contact lens sample procedure that followed real-life conditions of contact lens disposal after usage (it presents procedure ① in Figure 7).

Real-Life Conditions	Simulation in the Lab
**1. Contact lenses are removed from the eyes: getting dried**	
	Unused contact lenses in the case
**2. Rubbed off by the hands of the user**	
a. Getting dried state	Drying in the desiccator
b. Fragmentation	Grinding with a mortar and pestle
**3. Disposal to the toilet/basin/sewage system**	
a. Staying in aquatic environments	Immersed in DI water
**4. In the case of degradation of contact lens fragments under decomposing conditions**	
a. NPs generation by degradation	b. HCl treatment to accelerate decomposing conditions
